# A Novel Pathway for Porcine Epidemic Diarrhea Virus Transmission from Sows to Neonatal Piglets Mediated by Colostrum

**DOI:** 10.1128/jvi.00477-22

**Published:** 2022-06-27

**Authors:** Chen Yuan, Penghao Zhang, Peng Liu, Yuchen Li, Jianda Li, En Zhang, Yuxin Jin, Qian Yang

**Affiliations:** a MOE Joint International Research Laboratory of Animal Health and Food Safety, College of Veterinary Medicine, Nanjing Agricultural University, Nanjing, Jiangsu, People’s Republic of China; Loyola University Chicago

**Keywords:** colostrum, T cells, neonatal piglets, PEDV, mammary epithelial cells

## Abstract

The mechanisms of colostrum-mediated virus transmission are difficult to elucidate because of the absence of experimental animal models and the difficulties in tissue sample collection from mothers in the peripartum period. Porcine epidemic diarrhea virus (PEDV) is a reemerging enteropathogenic coronavirus that has catastrophic impacts on the global pig industry. PEDV primarily infects neonatal piglets by multiple routes, especially 1- to 2-day-old neonatal piglets. Here, our epidemiological investigation and animal challenge experiments revealed that PEDV could be vertically transmitted from sows to neonatal piglets via colostrum, and CD3^+^ T cells in the colostrum play an important role in this process. The results showed that PEDV colonizing the intestinal epithelial cells (IECs) of orally immunized infected sows could be transferred to CD3^+^ T cells located just beneath the IECs. Next, PEDV-carrying CD3^+^ T cells, with the expression of integrin α4β7 and CCR10, migrate from the intestine to the mammary gland through blood circulation. Arriving in the mammary gland, PEDV-carrying CD3^+^ T cells could be transported across mammary epithelial cells (MECs) into the lumen (colostrum), as illustrated by an autotransfusion assay and an MECs/T coculture system. The PEDV-carrying CD3^+^ T cells in colostrum could be interspersed between IECs of neonatal piglets, causing intestinal infection via cell-to-cell contact. Our study demonstrates for the first time that colostrum-derived CD3^+^ T cells comprise a potential route for the vertical transmission of PEDV.

**IMPORTANCE** The colostrum represents an important infection route for many viruses. Here, we demonstrate the vertical transmission of porcine epidemic diarrhea virus (PEDV) from sows to neonatal piglets via colostrum. PEDV colonizing the intestinal epithelial cells could transfer the virus to CD3^+^ T cells located in the sow intestine. The PEDV-carrying CD3^+^ T cells in the sow intestine, with the expression of integrin α4β7 and CCR10, arrive at the mammary gland through blood circulation and are transported across mammary epithelial cells into the lumen, finally leading to intestinal infection via cell-to-cell contact in neonatal piglets. Our study not only demonstrates an alternative route of PEDV infection but also provides an animal model of vertical transmission of human infectious disease.

## INTRODUCTION

Porcine epidemic diarrhea virus (PEDV) causes acute diarrhea, dehydration, and up to 100% mortality in neonatal piglets after birth (1–3). Currently, a safe and effective vaccine against PEDV infection in neonatal piglets is unavailable, making PEDV prevention and control challenging. Because of the high virulence of PEDV and the immature immune system of neonatal piglets, passive lactogenic immunity to PEDV is critical for neonatal piglet protection (4). In enteric virus-infected sows, gut-derived immunoglobulin A (IgA) immunocytes migrate to the mammary glands (MGs) and produce high titers of secretory IgA (sIgA) antibodies in the colostrum (via the gut-mammary-sIgA axis), contributing to passive lactogenic immunity (5, 6). Clinically, to control PEDV, involving “feedback” or controlled whole-herd exposure, entire sow herds are fed infectious material from their farm (i.e., feces and gut tissues collected from infected piglets) to stimulate active immunity against PEDV via the gut-mammary axis (4). The immunity developed against PEDV in sows could then be transferred to neonatal piglets through colostrum/milk. The outcome of this strategy has been satisfactory because the incidence of clinical disease has markedly declined; however, feedback has not been 100% effective (7). The sows were actually immunized with live vaccines, whereas their piglets were still clinically infected with PEDV. However, the underlying mechanisms by which PEDV leads to infection in neonatal piglets have not been confirmed.

Recently, based on decreased neonatal piglet death rates as a result of fostering, many researchers have highlighted the possible role of colostrum transmission of PEDV. It has been reported that PEDV not only can be detected in the colostrum but also can be isolated from the colostrum samples of immunized infected sows on pig farms (2, 8). However, the origin of PEDV in the colostrum remains unclear.

In the present study, the hypothesis that sow colostrum could represent a novel route for PEDV transmission from sows to suckling piglets is proposed. Both experiments with validated animal models and reported clinical evidence confirmed that neonatal piglets could be infected with PEDV via the colostrum. Coculture models were established *in vitro* using mammary epithelial cells (MECs) and T lymphocytes, as well as T lymphocytes and Vero cells, to gain more insight into the mechanism of PEDV entry into the intestinal epithelium in neonatal piglets via the colostrum from immunized infected sows. To the best of our knowledge, our study confirmed for the first time that colostrum-derived CD3^+^ T cells are a potential route for the vertical transmission of PEDV from sows to neonatal piglets.

## RESULTS

### Transmission of PEDV from clinically immunized infected mothers to breastfed neonates.

Recently, our epidemiological investigation showed that sows and suckling neonatal piglets exhibited different degrees of diarrhea and no appetite after sows were immunized by being fed PEDV infectious material from their farms; however, the degree of diarrhea decreased significantly if neonatal piglets were prevented from suckling. This phenomenon prompted our interest in studying what caused diarrhea in neonatal piglets.

**FIG 1:**
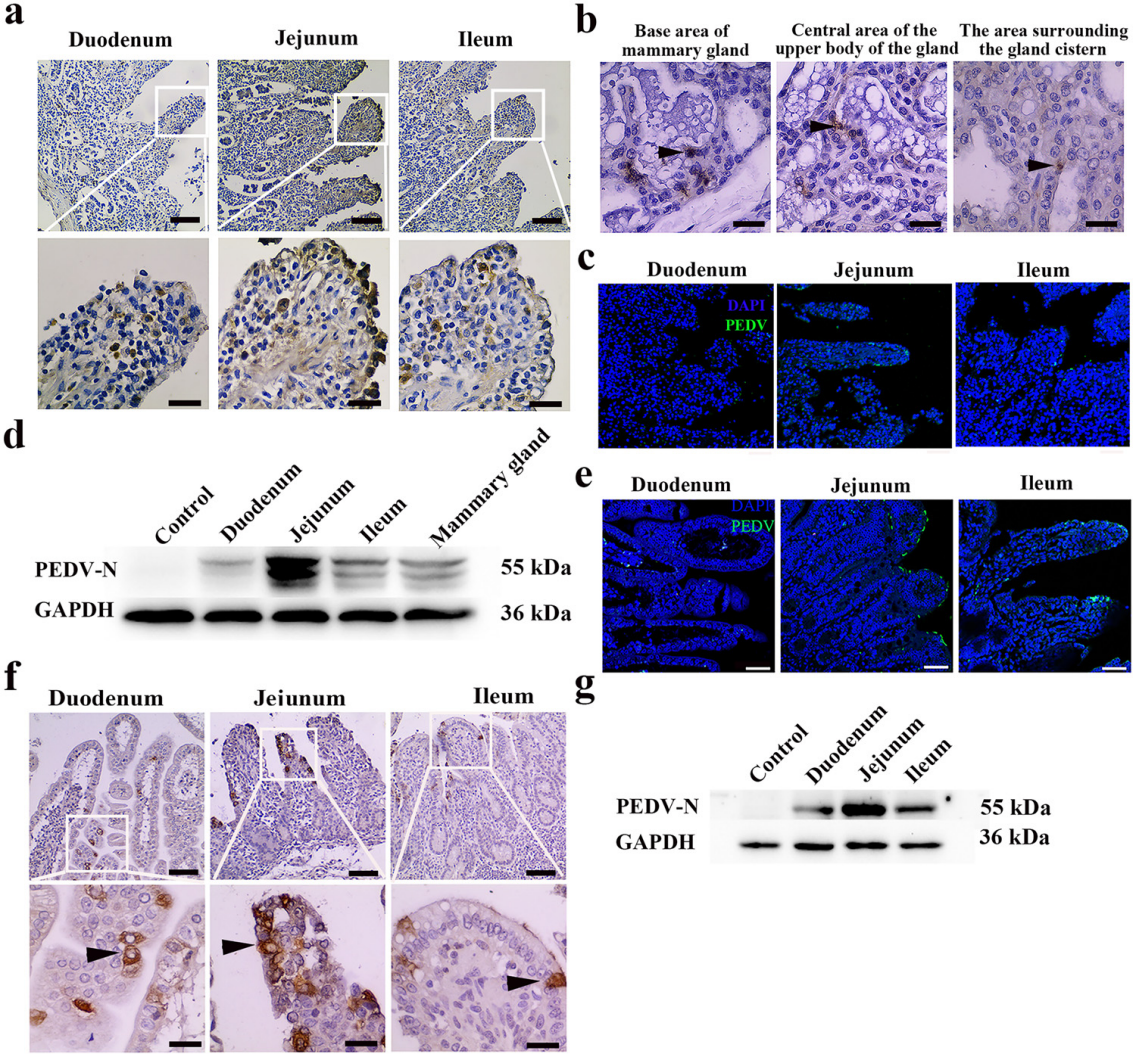
Distribution of PEDV in sows and neonatal piglets. All sows, a total of 1,000, including gilts, are fed infected material at one time, except for sows in the farrowing house (within 21 days before delivery). The sows in this study were fed PEDV feedback material at about 60 days of gestation and euthanized 72 h after delivery to collect tissue for testing. (a) IHC of the intestine of an immunized infected sow during lactation. PEDV antigen was located in the intestinal villus. Bars = 100 μm (top) and 20 μm (bottom). (b) IHC of the MG of immunized infected sows. PEDV antigen was located in the breast. Bars = 20 μm. (c) IFA of the intestine of the immunized infected sows. PEDV antigen was located in the intestinal villus. Bars = 50 μm. Blue, DAPI; green, PEDV. (d) Protein expression of PEDV in different tissues of the immunized infected sows during lactation as determined by Western blotting with a mouse mAb against N protein. (e) IFA of the intestine of suckling neonatal piglets with diarrhea. PEDV antigen was located in the intestinal villus. Bars = 50 μm. Blue, DAPI; green, PEDV. (f) IHC of the intestines of suckling neonatal piglets with diarrhea. Bars = 100 μm (top) and 20 μm (bottom). (g) Protein expression of PEDV in the intestine of neonatal piglets with diarrhea was determined by Western blotting with a mouse mAb against N protein. Bars = 50 μm. Blue, DAPI; green, PEDV.

First, we sought to detect the presence of PEDV in immunized infected sows and their neonatal piglets from farms with diarrhea. Immunohistochemistry (IHC) staining and immunofluorescence analysis (IFA) results showed that many PEDV-positive (PEDV^+^) cells were observed in the small intestine and MG of immunized infected sows (Fig. 1a to c). Western blot analysis results further validated the PEDV levels in different tissues, and a significant quantity of PEDV N protein was detected in the jejunum and MG of immunized infected sows (Fig. 1d). Next, the distribution of PEDV in the small intestine of neonatal piglets was determined by a variety of methods. As described below, PEDV could also be detected in the duodenum, jejunum, and ileum of neonatal piglets breastfed by immunized infected sows (Fig. 1e to 1g). These results suggested that neonatal piglets may be clinically infected with PEDV via colostrum.

### Virus-carrying T cells in the colostrum cause PEDV infection in neonatal piglets.

**FIG 2:**
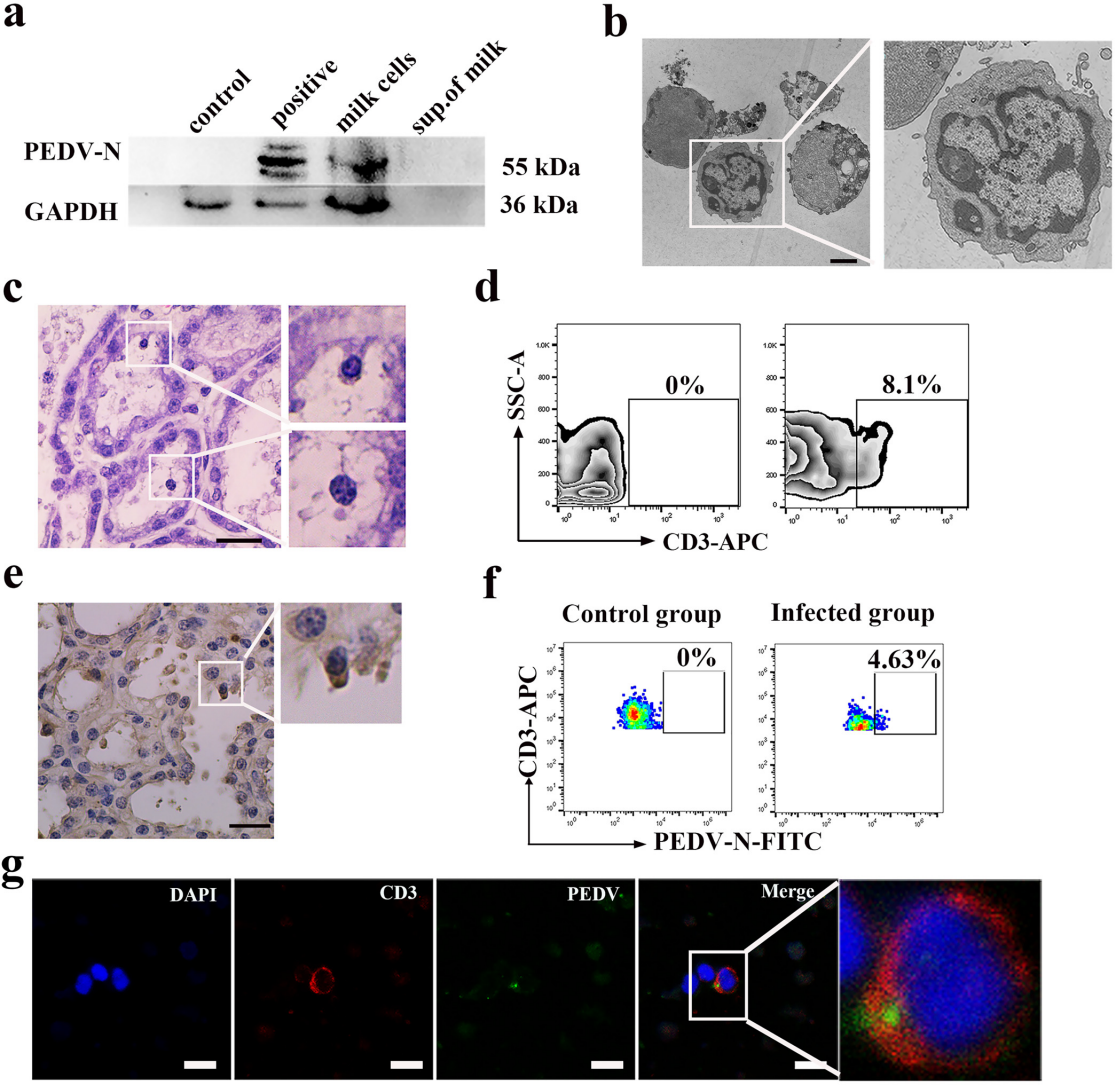
CD3^+^ T cells carrying PEDV were identified in the colostrum of immunized infected sows. The results are from one sow as a representative example. Twelve PEDV-positive milk samples were tested by different methods. (a) Protein expression of PEDV in colostrum from an immunized infected sow was determined by Western blotting with a mouse mAb against N protein. (b) Lymphocytes in colostrum were identified by transmission electron microscopy (TEM). Bar = 2 μm. (c) Hematoxylin and eosin (H&E) staining of MG tissue of the immunized infected sow. The enlarged cells are lymphocytes. Bar = 20 μm. (d) CD3^+^ T cells carrying PEDV from sow colostrum were analyzed by FACS. SSC, side scatter. (e) IHC showing CD3^+^ T cells in the MG. The enlarged image represents CD3^+^ T cells. Bar = 20 μm. (f and g) FACS analysis (f) and IFA (g) examining PEDV in CD3^+^ T cells of colostrum collected from the immunized infected sow during lactation. Bars = 10 μm. Blue, DAPI; green, PEDV; red, CD3. FITC, fluorescein isothiocyanate.

To investigate how neonatal piglets could become infected with PEDV via colostrum, we conducted an experimental survey by collecting a total of 200 samples (colostrum) from sows diagnosed with diarrhea on four farms. The N gene of PEDV could be detected by PCR in 44.5% (89/200) of sow colostrum samples (data not shown). Western blot analysis revealed significant PEDV N protein quantities in breast milk cells, but no PEDV N protein was detected in the milk supernatant (Fig. 2a). We suspected that some cell types within the colostrum could carry PEDV. It was previously reported by our laboratory that CD3^+^ T cells could carry PEDV for viral infection in piglets (3). Thus, we employed various methods to identify CD3^+^ T cells in colostrum. As expected, CD3^+^ T cells not only were distributed in MGs but also could be transported across mammary epithelial cells (MECs) into the lumen (colostrum) (Fig. 2b to e). To our surprise, fluorescence-activated cell sorting (FACS) analysis revealed that 4.63% of the CD3^+^ T cells in the colostrum from immunized infected sows were PEDV positive (Fig. 2f). Furthermore, we used confocal microscopy to visualize the subcellular locations of PEDV and CD3^+^ T cells. IFA showed that PEDV N protein was colocalized with CD3^+^ T cells and that PEDV was indeed internalized by the CD3^+^ T cells (Fig. 2g).

**FIG 3:**
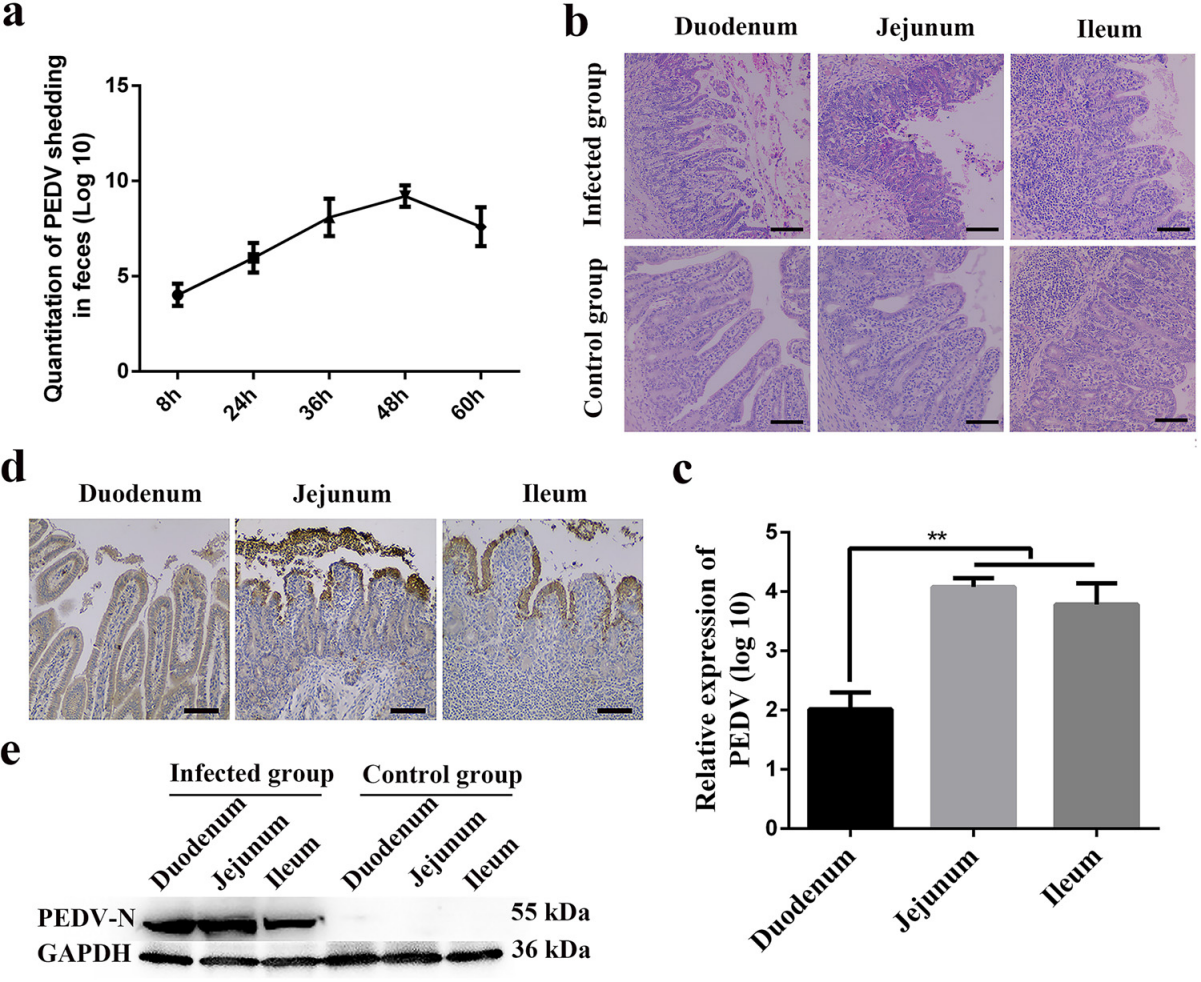
CD3^+^ T cells carrying virus in the colostrum caused PEDV infection in neonatal piglets. (a) Titers of PEDV RNA shedding PEDV RNA in the feces were determined by RT-qPCR. (b) Villus morphology of neonatal piglets in the control group and the infected group under light microscopy. (c) Viral RNA expression in the small intestine of the diarrheic piglets after oral inoculation with CD3^+^ T cells carrying PEDV (3 piglets per group). (d) IHC of the intestine of piglets after oral inoculation with CD3^+^ T cells carrying PEDV. PEDV antigen was located in the intestinal villus. Bars = 20 μm. (e) Protein expression of PEDV in the small intestine of diarrheic piglets was determined by Western blotting with a mouse mAb against N protein. At least three independent experiments were performed. All data represent the means ± SD. Comparisons were performed by analysis of variance (ANOVA) (multiple groups). *, *P < *0.05; **, *P < *0.01. Data were combined from at least three independent experiments.

To evaluate the pathogenicity of PEDV particles in colostrum-derived cells, animal experiments were conducted. First, the results showed that a large number of viruses are distributed in the small intestine of neonatal piglets after being orally administered colostrum-derived cells from immunized infected sows (data not shown). Subsequently, to determine whether PEDV-carrying CD3^+^ T cells from colostrum were sufficient to cause infection of neonatal piglets, we also carried out animal challenge experiments. Colostrum-derived CD3^+^ T cells from immunized infected sows were isolated by magnetic bead separation (magnetically activated cell sorting [MACS]) and directly administered to neonatal piglets. Of course, the colostrum-derived CD3^+^ T cells acquired were active. Piglets in the control groups were fed an equal volume of phosphate-buffered saline (PBS). Neonatal piglets that were orally administered the CD3^+^ T cells of colostrum began to exhibit white foam vomit at 8 h postinfection (hpi), which lasted for about 1 day. At 30 hpi, the neonatal piglets began to exhibit diarrhea, and their feces were gray-yellow and watery. At 60 hpi, diarrhea of different degrees occurred, with large, yellow, foul-smelling watery stools. Quantitation of PEDV shedding in the feces of neonatal piglets orally administered colostrum-derived CD3^+^ T cells from immunized infected sows was performed by real time-quantitative PCR (RT-qPCR) at different times. The results showed that PEDV gene copies reached a peak of 9.95 log_10_ copies within 48 hpi and then decreased gradually (Fig. 3a). Finally, the inoculated and negative-control piglets were euthanized for pathogen examination at 60 hpi. Compared to neonatal piglets in the control group, the height of the intestinal villi in infected piglets was significantly reduced, with hemorrhage and shedding of villous epithelial cells (Fig. 3b). RT-PCR, IHC, and Western blotting results revealed PEDV in the duodenum, jejunum, and ileum of piglets after the oral administration of CD3^+^ T cells from the colostrum (Fig. 3c to e). Therefore, our results confirmed that colostrum-derived T cells from immunized infected sows could cause PEDV infection in neonatal piglets.

### T cells acquire PEDV from intestinal epithelial cells and migrate to the mammary gland via blood circulation in sows.

**FIG 4:**
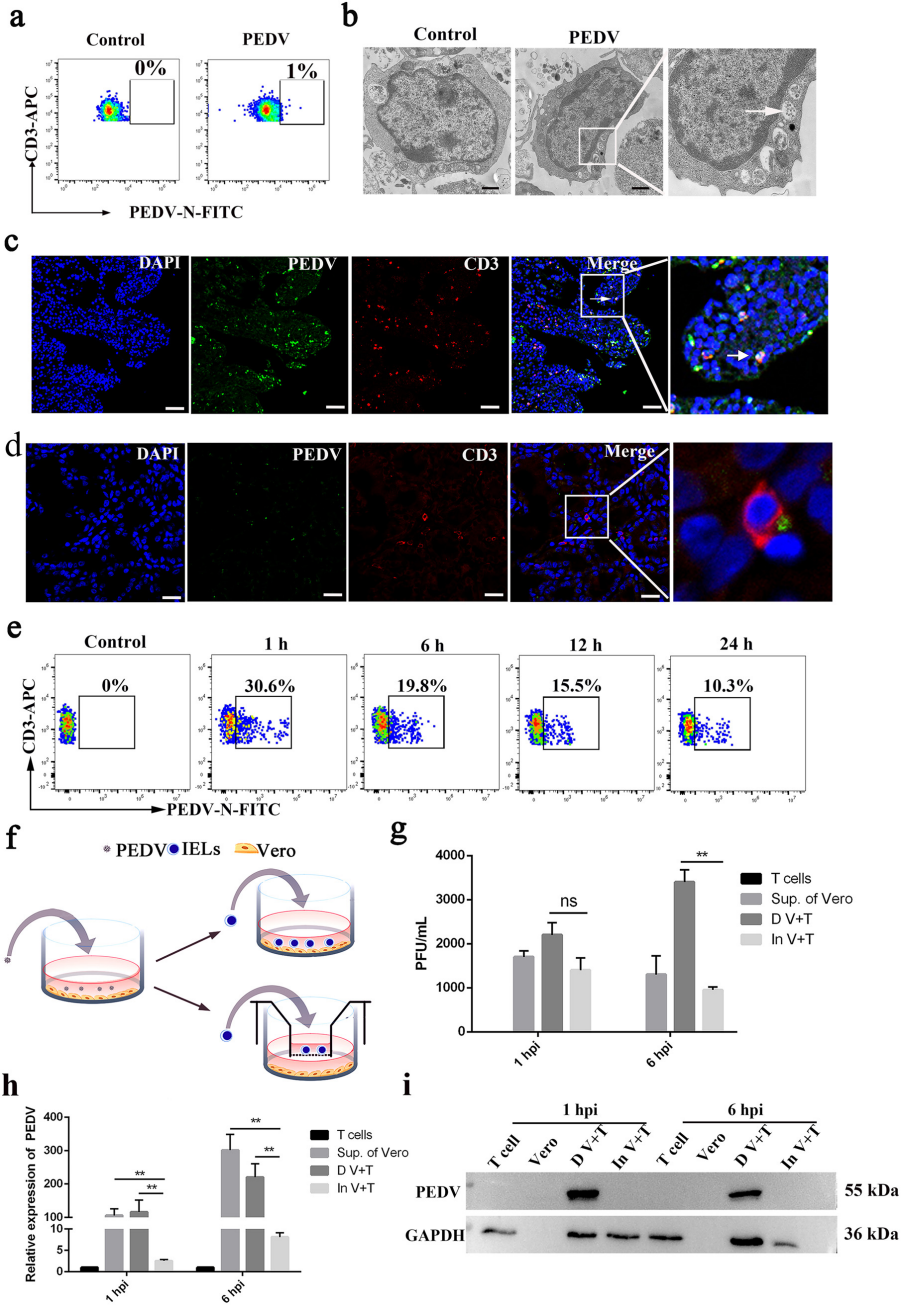
CD3^+^ T cells acquire PEDV from intestinal epithelial cells (IECs) and migrate to MGs in sows. (a) FACS analysis of PEDV^+^ CD3^+^ T cells in PBMCs from immunized infected sows. (b) TEM image showing the morphological features and PEDV of the CD3^+^ T cells in PBMCs from a sow infected with PEDV. Bars = 1 μm. (c and d) Frozen sections of the jejunum (c) and breast (d) from a sow infected with PEDV during lactation were stained with different antigens and observed by fluorescence microscopy. PEDV^+^ CD3^+^ T cells were located in the intestinal villus and MG. Bars = 50 μm. Blue, DAPI; green, PEDV; red, CD3. (e) Blood-derived CD3^+^ T cells were infected with PEDV at different times. The viral loads in blood-derived CD3^+^ T cells were determined by FACS. (f) Schematic of the model to study PEDV-carrying Vero cell transmission of the virus to T cells. T cells were cocultured with Vero cells by contact and noncontact cocultures. The contact coculture is the coincubation of virus-carrying Vero cells with T cells on cell plates. The noncontact coculture is T cells placed in the upper compartment of a transwell and cocultured with virus-carrying Vero cells indirectly. T cells were collected after at 1 and 6 hpi in coculture. PEDV of T cells could be detected in the early stage of coculture. (g) Viral titers in T cells of the coculture system were measured by a plaque assay (*n* = 3). “D V+T” represents direct contact between T cells and Vero cells. “In V+T” represents indirect contact between T cells and Vero cells. (h) Viral RNA expression in T cells of the coculture system at 1 h and 6 h (*n* = 3). (i) Protein expression of PEDV in T cells of the coculture system was detected by Western blotting with a mouse mAb against N protein. All data represent the means ± SD. Comparisons were performed by analysis of variance (ANOVA) (multiple groups). *, *P < *0.05; **, *P < *0.01; ns, not significant. Data were combined from at least three independent experiments.

PEDV infection is localized mainly to intestinal epithelial cells (IECs) of sows; however, our results revealed that there were T cells carrying PEDV in colostrum. Moreover, gut-derived immunocytes could utilize the gut-mammary axis to migrate to the mammary glands via blood circulation. Indeed, as shown in Fig. 4a, 1% of PEDV-carrying CD3^+^ T cells were detected in peripheral blood mononuclear cells (PBMCs) of immunized infected sows. Compared to the control, CD3^+^ T cells of the infected group exhibited several morphological variations such as cell elongation and the observed accumulation of cytoplasmic PEDV-like particles based on the size of the particles (95 to 190 nm) (Fig. 4b). Furthermore, IFA showed that PEDV was also present in CD3^+^ T cells in the intestine and mammary tissues of immunized infected sows *in vivo* (Fig. 4c and d). In order to further determine the viral titer in CD3^+^ T cells derived from the blood of sows infected by PEDV *in vitro*, CD3^+^ T cells derived from sow blood were inoculated with PEDV, and at 1, 6, 12, and 24 hpi, the CD3^+^ T cells were collected for FACS. The results showed that CD3^+^ T cells inoculated with PEDV reached a maximum of 30.6% at 1 hpi. The percentage of PEDV-positive CD3^+^ T cells gradually decreased from 19.8% to 10.3% over 6 to 24 hpi *in vitro* (Fig. 4e). These results indicated that CD3^+^ T cells might acquire the virus from IECs, enter PBMC circulation, and migrate to the mammary gland.

To explore how CD3^+^ T cells located beneath the IECs acquire PEDV from IECs, we next cocultured epithelial (Vero) and T cells in two different ways, namely, under contact and noncontact conditions, to study PEDV transmission from Vero cells to T cells *in vitro* (Fig. 4f). T cells were collected 1 and 6 h after infection in coculture. In contrast to T cells in the noncontact group, those in the group in contact with Vero cells exhibited higher PEDV RNA expression levels and virus titers (Fig. 4g and h). Western blot results further validated that PEDV loaded on Vero cells could be transferred to T cells mainly via direct contact (Fig. 4i). Our results demonstrated that PEDV could be transferred to T cells beneath the IECs in immunized infected sows and that T cells carrying PEDV could migrate to the MG via blood circulation.

### PEDV-carrying T cells migrate and are transported across mammary epithelial cells into the lumen (colostrum) in sows.

**FIG 5:**
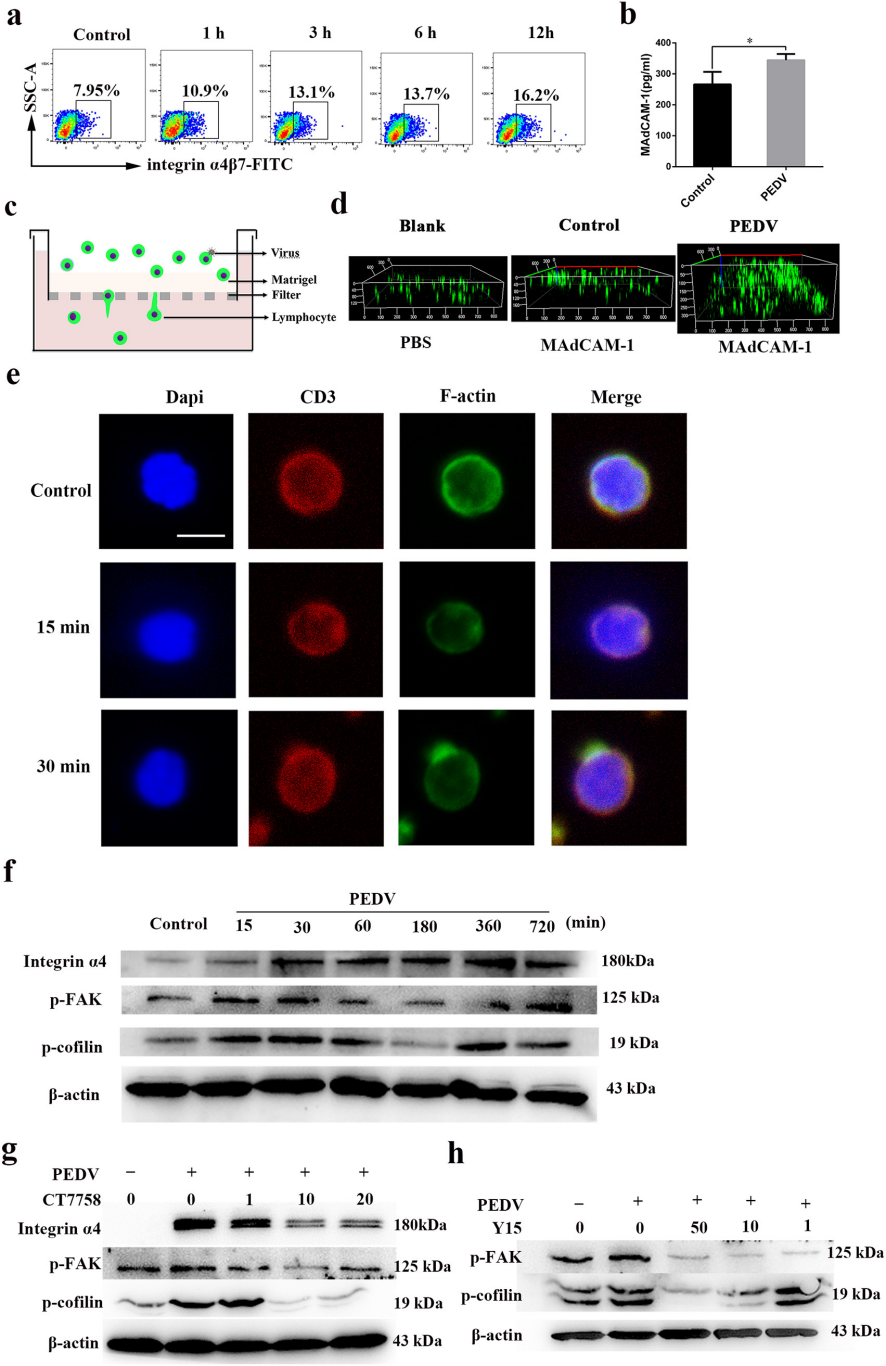
Integrin α4β7 is involved in PDEV-carrying CD3^+^ T cell migration from the intestine to the mammary gland. (a) T cells were infected with PEDV at different times. The expression of the phenotypic marker integrin α4β7 on T cells was analyzed by FACS (*n* = 3 per group). (b) The levels of MAdCAM-1 in colostrum from sows (control and infected) were determined by an ELISA. (c) Schematic representation of the transwell migration assay. (d) The lower sides of 8-μm-pore-size polycarbonate filters of a 24-well transwell plate were coated with 100 μL of 50% Matrigel. CD3^+^ T cells or CD3^+^ T cells infected with PEDV (10^6^ cells/mL) were added to the upper chamber in the presence or absence of the chemokine MAdCAM-1 overexpressed by prokaryotic expression in the lower chamber. At 12 hpi, images of migrated CD3^+^ T cells were taken using a Zeiss LSM710 confocal microscope. (e) F-actin reorganization of CD3^+^ T cells after PEDV infection. The CD3^+^ T cells were infected with PEDV (MOI = 1) for 0, 15, or 30 min and then fixed, permeabilized, and stained with CD3 antibody, TRITC-phalloidin, and DAPI. Images were then recorded with a confocal laser scanning microscope. (f) Time course of integrin α4, FAK phosphorylation, and cofilin phosphorylation following PEDV infection. T cells were infected with PEDV at an MOI of 1, and at the indicated time points, the cell lysates were prepared for Western blotting. (g and h) T cells were pretreated with the integrin α4 inhibitor CT7758 (g) or the FAK inhibitor Y15 (h) at the indicated concentrations for 1 h and then infected with PEDV (MOI = 1) in the presence of the respective inhibitors for 0.5 h. The cell lysate was prepared and subjected to Western blotting.

Integrin α4β7 on T cells, interacting with mucosal addressin cellular adhesion molecule 1 (MAdCAM-1), plays an important role in the migration of T cells from the intestine to the MG in swine (4). We estimated the level of integrin α4β7 expression on the surface of T cells. Indeed, FACS analysis indicated a high level of integrin α4β7 expression in PEDV-infected T cells at different time points (Fig. 5a). In addition, compared to that in the controls, the MAdCAM-1 protein level in the colostrum was upregulated in immunized infected sows (Fig. 5b). The effect on T cell migration was evaluated using Matrigel-coated polycarbonate membranes (Fig. 5c). The chemokine MAdCAM-1 increased CD3^+^ T cell migration at 500 ng/mL at 24 hpi in the PEDV-infected group (Fig. 5d), thus confirming that T cell migration is mediated mostly by interactions between surface integrin α4β7 and MAdCAM-1.

To confirm that PEDV could alter the surface actin filament-based structure, we performed fluorescent tetramethyl rhodamine isothiocyanate (TRITC)-phalloidin-labeled F-actin staining in the presence or absence of PEDV. Compared to the controls, cytoskeleton variation, the aggregation of F-actin at the margins of cells, was observed in infected CD3^+^ T cells at 15 and 30 min (Fig. 5e). Moreover, the levels of integrin α4β7 and phosphorylated focal adhesion kinase (FAK) and cofilin in infected CD3^+^ T cells were significantly increased upon PEDV infection (Fig. 5f). A previous study demonstrated that cofilin and FAK signaling can be activated by integrin α4β7, suggesting that PEDV might activate cofilin and FAK signaling through integrin α4β7 (9). Therefore, we examined the effects of the integrin α4β7 inhibitor CT7758 on cofilin and FAK signaling in response to PEDV infection. As expected, the PEDV-induced activation of cofilin and FAK was attenuated by the CT7758-dependent inhibition of integrin α4β7 (Fig. 5g). To further investigate whether PEDV infection activated cofilin signaling through FAK signaling, we examined the effects of the inhibitor Y15 on FAK. The results showed that the inhibitor Y15 decreased the levels of phosphorylated FAK, and similarly, the PEDV-induced activation of cofilin was attenuated in a Y15-dose-dependent manner (Fig. 5h). Therefore, our results indicated that PEDV utilizes the integrin α4β7-FAK-cofilin pathway to cause actin cytoskeletal rearrangement in CD3^+^ T cells.

**FIG 6:**
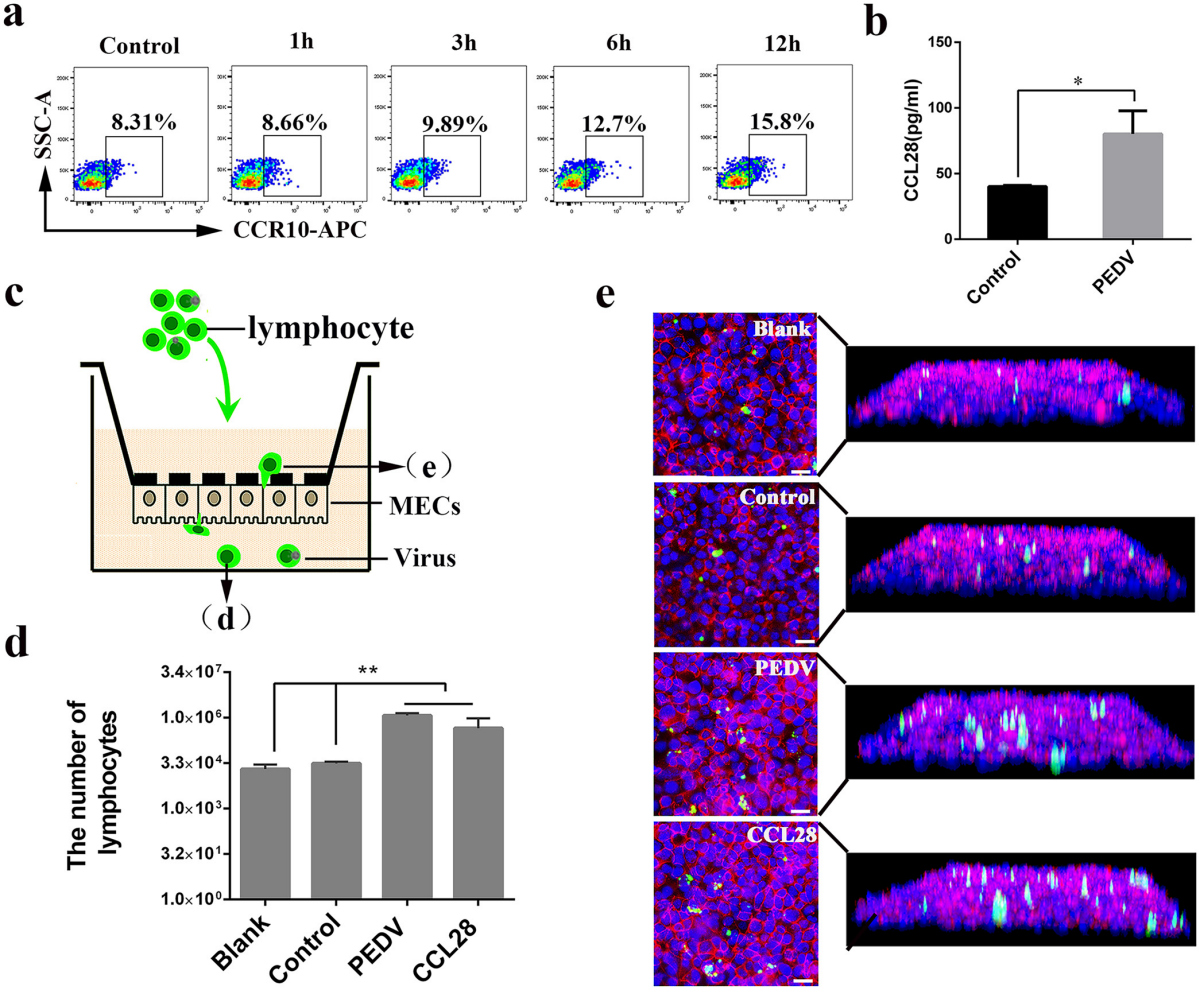
CCR10 is involved in the transport of PEDV-carrying CD3^+^ T cells across mammary epithelial cells into the lumen (colostrum). (a) T cells were infected with PEDV at different times. The expression of the phenotypic marker CCR10 on T cells was analyzed by FACS (*n* = 3 per group). (b) The levels of CCL28 in colostrum from sows (control and infected) were determined by an ELISA. (c) Schematic representation of the transwell migration assay. Fluorescently labeled lymphocytes (10^6^ cells/mL) infected with PEDV or the controls are added to the upper chamber in the presence or absence of chemokines or colostrum in the lower chamber. (d) Counting of cells retrieved from the lower chamber enables the determination of the number of lymphocytes that fully migrated through the mammary epithelial cells. (e) Protocols for staining of the cell-covered filters following the migration study to allow defining the localization of lymphocytes within the filter/epithelium system and approaching the route of transmigration. Bars = 20 μm. Blank, fluorescently labeled lymphocytes and blank medium in the upper chamber and the lower chamber, respectively; Control, fluorescently labeled lymphocytes infected with PEDV and colostrum acquired from healthy sows in the upper chamber and the lower chamber, respectively; PEDV, fluorescently labeled lymphocytes infected with PEDV and colostrum acquired from infected sows in the upper chamber and the lower chamber, respectively; CCL28, fluorescently labeled lymphocytes infected with PEDV and the chemokine CCL28 in the upper chamber and the lower chamber, respectively.

T cells recruited by the MG must pass through the mammary epithelium (MECs) into the lumen. As shown in Fig. 6a, a high level of CCR10 was detected in PEDV-infected CD3^+^ T cells at different times. In addition, unlike in the controls, CCL28 was upregulated at the protein level in the milk of immunized infected sows (Fig. 6b). Therefore, we speculated that the passage of PEDV-carrying T cells into the lumen through MECs depended on the interaction between the surface chemokines CCR10 and CCL28. An MEC-T cell coculture system was used to study T cell migration (Fig. 6c). Inoculation with colostrum-containing live PEDV increased the number of carboxyfluorescein succinimidyl amino ester (CFSE)-labeled T cells compared to that with inoculation with control colostrum (Fig. 6d). Of note, neither T cell coculture nor PEDV challenge modified the morphological characteristics of tight junctions in MECs (data not shown). Moreover, IFA confirmed the crossing of PEDV-carrying CD3^+^ T cells through the MECs into the lumen of the MG (Fig. 6e). Our results revealed that the migration and passage of PEDV-carrying T cells through MECs into the lumen (colostrum) are mediated mostly by interactions between surface integrin α4β7 and CCR10 on T cells and between MAdCAM-1 and CCL28 on MECs.

### PEDV-carrying T cells in the colostrum could transfer the virus to intestinal epithelial cells of neonatal piglets.

**FIG 7:**
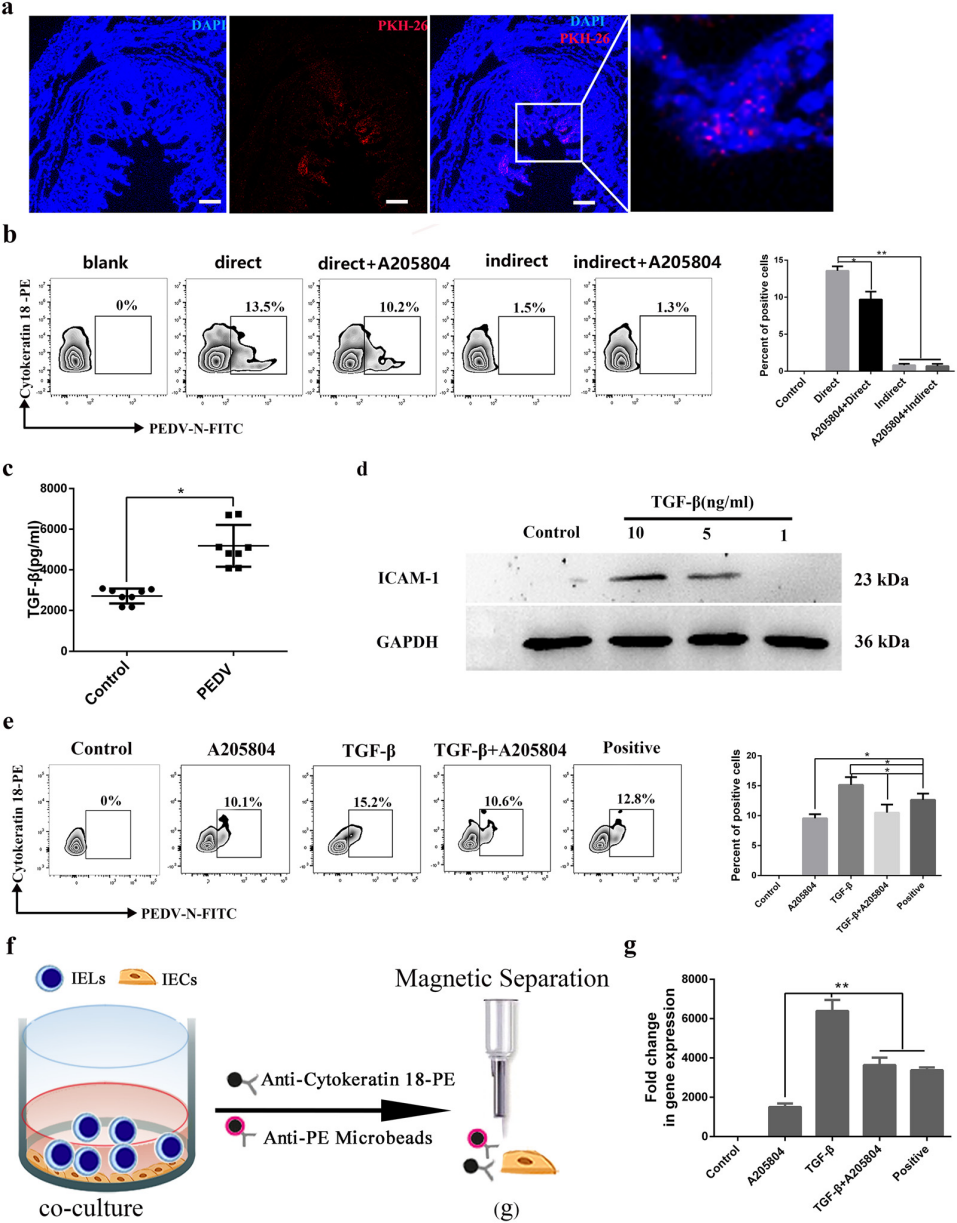
Role of TGF-β in T cell-to-IEC contact-mediated PEDV transmission. (a) CD3^+^ T cells were collected from colostrum from a sow that had just delivered. The CD3^+^ T cells were then labeled with the fluorescent marker PKH26 and fed to the neonatal piglets. After 12 h, the piglets were anesthetized with pentobarbital sodium (100 mg/kg). Frozen sections of the jejunum from the groups fed fluorescence-labeled cells were stained with DAPI (blue) and observed by fluorescence microscopy. Bars = 50 μm. (b) Model to study PEDV-carrying CD3^+^ T cell transmission of the virus to intestinal epithelial cells (IECs). PEDV-carrying CD3^+^ T cells were cocultured with IECs by two methods (contact and noncontact cocultures) for 3 h and cocultured with or without the ICAM-1 inhibitor A205804 (50 ng/mL). Cells were collected from the coculture system, and cytokeratin 18 were detected by FACS. (c) The levels of TGF-β in colostrum from sows (control and infected) were determined by an ELISA. (d) IECs sorted by MACS were cultured for 2 h with or without TGF-β. The levels of ICAM-1 and GAPDH were determined by Western blotting. (e) IECs were treated with or without TGF-β at a concentration of 5 ng/mL. PEDV-pulsed CD3^+^ T cells were cocultured with IECs by contact methods for 3 h and cocultured with or without the ICAM-1 inhibitor A205804. The cells were collected from the coculture system, and IECs and cytokeratin 18 were detected by FACS. (f) IECs were treated with or without TGF-β. PEDV-pulsed CD3^+^ T cells were cocultured with IECs by contact methods for 3 h and cocultured with or without the ICAM-1 inhibitor A205804. IECs marked with cytokeratin 18 were isolated by MACS. (g) The gene levels of virus in IECs from PEDV-pulsed CD3^+^ T cells cocultured with IECs were determined by RT-PCR. All data represent the means ± SD. Comparisons were performed by analysis of variance (ANOVA) (multiple groups). *, *P < *0.05; **, *P < *0.01. Data were combined from at least three independent experiments.

To explore the mechanism by which CD3^+^ T cells carrying PEDV within colostrum caused PEDV infection in neonatal piglets, CD3^+^ T cells of colostrum from immunized infected sows were isolated, labeled with PKH26, (PKH26) and directly administered to neonatal piglets. IFA results revealed that colostrum-derived T cells could be interspersed between the IECs of neonatal piglets. Thus, cell-to-cell contact structures provided an opportunity for virus transmission between T cells of the colostrum and IECs of neonatal piglets (Fig. 7a). To further investigate whether viral transmission occurs between CD3^+^ T cells and IECs of neonatal piglets, CD3^+^ T cells and IECs were isolated from sow’s blood and the intestine of neonatal piglets, respectively. A T cell-IEC coculture system was established in two forms, contact and noncontact. FACS results showed that as much as 13.5% of PEDV^+^ cytokeratin 18-positive (CK18^+^) IECs were detected in the contact group at 3 h, which was much higher than that in the noncontact group. These results indicated that PEDV-carrying T cells allowed the virus to be transferred to IECs mainly via direct contact. When IECs were treated with the ICAM-1 inhibitor A205804, many PEDV-containing IECs were significantly reduced in the coculture system (Fig. 7b). These data demonstrated that ICAM-1 plays a positive role in PEDV transmission between CD3^+^ T cells and IECs.

Maternal cytokines and antibodies in the colostrum could affect the function of IECs in neonatal piglets. Therefore, we investigated the concentrations of interleukin-6 (IL-6) and tumor necrosis factor alpha (TNF-α) (proinflammatory), interferon gamma (IFN-γ) and IL-12 (Th1), IL-10 and IL-4 (Th2), transforming growth factor β1 (TGF-β1) (Th3), IgA, and IgG in the colostrum of healthy sows and sick sows via an enzyme-linked immunosorbent assay (ELISA). The level of PEDV-specific IgA in the colostrum was significantly decreased during infection, but there was no significant difference in IgG antibodies in serum. Surprisingly, the level of the cytokine TGF-β was significantly elevated in immunized infected sows compared to the levels in the control group, whereas the other cytokines were associated with the opposite results, except for IL-12 and IL-4, which were not significantly different (Fig. 7c and data not shown). IECs treated with 5 or 10 ng/mL of TGF-β for 2 h increased the expression of ICAM-1 in comparison to that in the control samples (Fig. 7d). To determine whether TGF-β could promote viral transmission between CD3^+^ T cells and IECs by upregulating the expression of ICAM-1 on IECs, a coculture system between CD3^+^ T cells and IECs was established. *In vitro* estimation by FACS analysis showed that TGF-β at a concentration of 5 ng/mL could significantly increase the virus yield in IECs. However, the ICAM-1 inhibitor A205804 inhibited viral transmission between CD3^+^ T cells and IECs (Fig. 7e). Moreover, IECs from the coculture model were isolated using a MACS separator column, and viral genes in IECs were detected using RT-PCR (Fig. 7f). The detection of the viral genes in IECs confirmed the FACS results (Fig. 7g). These results indicated that TGF-β promotes viral transmission between CD3^+^ T cells and IECs by upregulating the expression of ICAM-1 in IECs.

### Development of animal models for verification of PEDV-carrying T cells arriving at the MG in sows.

**FIG 8:**
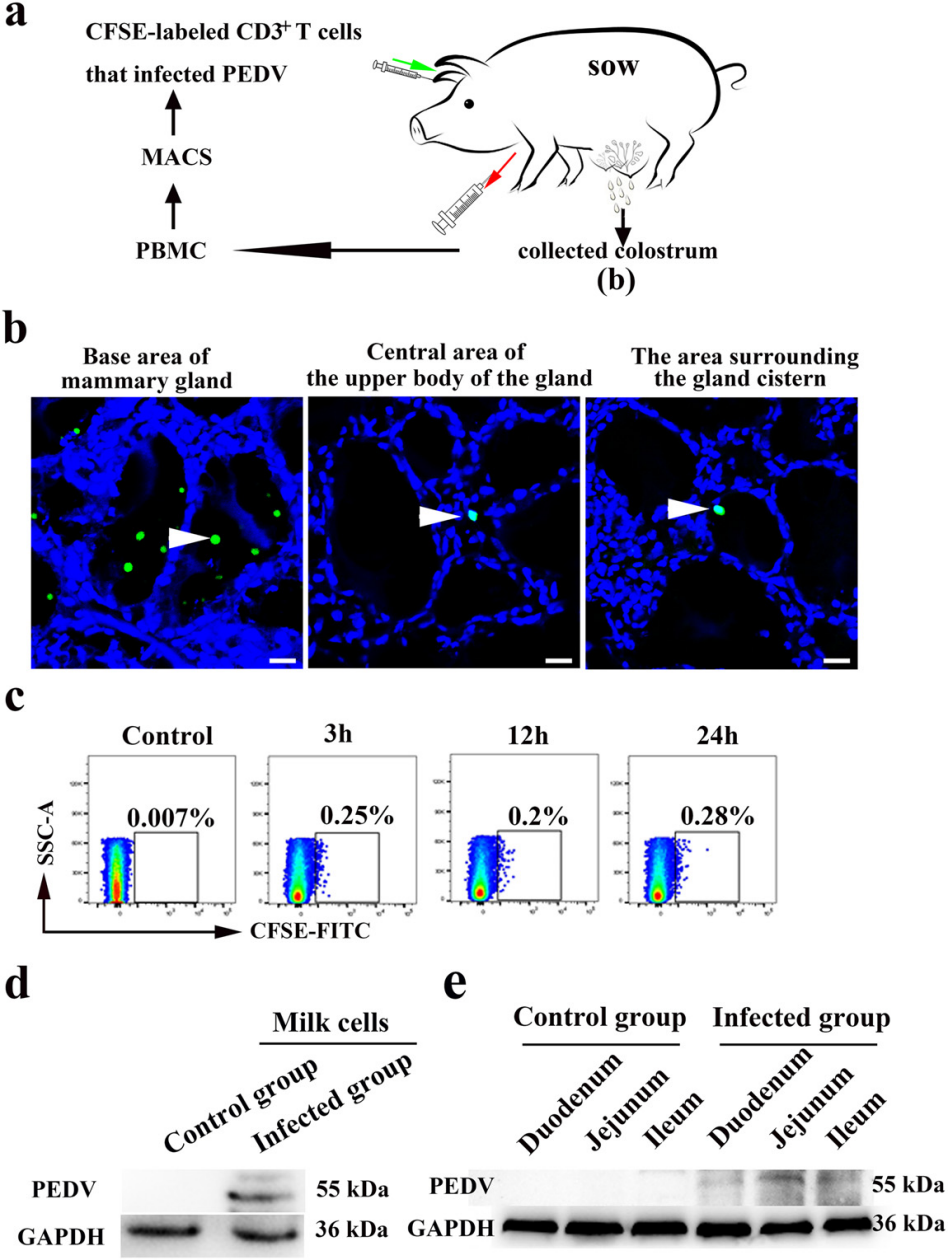
Development of animal models for verification of CD3^+^ T cells carrying PEDV arriving at the MG in sows. (a) Schematic representation of the model to study PEDV-carrying CD3^+^ T cells crossing mammary epithelial cells and reaching the colostrum. After sow delivery, 150 mL of blood was collected from the front cavity vein. PBMCs were isolated from the blood by density centrifugation using a porcine peripheral blood lymphocyte separation kit. CD3^+^ T cells were isolated from PBMCs by MACS and labeled with CFSE. CFSE-labeled CD3^+^ T cells that were infected with PEDV were resuspended in 1 mL of phosphate-buffered saline (PBS) and injected into sow blood via the ear vein. (b) After the *in vivo* autotransfusion assay, frozen sections of the MG, including the base area of the mammary gland (AB), the central area of the upper body of the gland (CAUB), and the area surrounding the gland cistern (ASGC), from sows injected with fluorescently CFSE-labeled cells were stained with DAPI (blue) and observed by fluorescence microscopy. CD3^+^ T cells labeled with CFSE are marked with white arrowheads. Bars = 20 μm. (c) For the *in vivo* autotransfusion assay, 20 mL of colostrum was collected into a 50-mL tube at different times. The cells in the colostrum were washed twice and isolated with ice-cold PBS. Cells labeled with CFSE were detected by FACS. (d) After a 24-h *in vivo* autotransfusion assay, the protein expression of PEDV in cells from colostrum was determined by Western blotting with a mouse mAb against N protein. (e) Twenty-four hours after birth, the protein expression of PEDV in the small intestine of neonatal piglets breastfed by sows was determined by Western blotting with a mouse mAb against N protein.

To evaluate whether PEDV-loaded CD3^+^ T cells in the blood could reach the mammary gland and cause infection in neonatal piglets via colostrum, we performed a PEDV-carrying CD3^+^ T cell reinfusion assay in sows (Fig. 8a). After 24 h, CFSE-labeled CD3^+^ T cells were found in the base area of the MG (AB), the central area of the upper body of the gland (CAUB), and the area surrounding the gland cistern (ASGC). More CFSE-labeled T cells were distributed in the AB than in the CAUB and ASGC of the MG (Fig. 8b). Furthermore, FACS results showed that CFSE-labeled T cells could be detected in the colostrum after 3, 12, and 24 h (Fig. 8c). A visible band was observed in colostrum from sows injected with PEDV-infected CD3^+^ T cells after 24 h. However, no apparent bands were observed in control sow samples (Fig. 8d). PEDV could also be detected in the duodenum, jejunum, and ileum of breastfed neonatal piglets (Fig. 8e). Therefore, our results demonstrated that CD3^+^ T cells carrying PEDV could migrate to the MG via blood circulation and be transported across MECs into the lumen, causing PEDV infection in neonatal piglets.

## DISCUSSION

**FIG 9:**
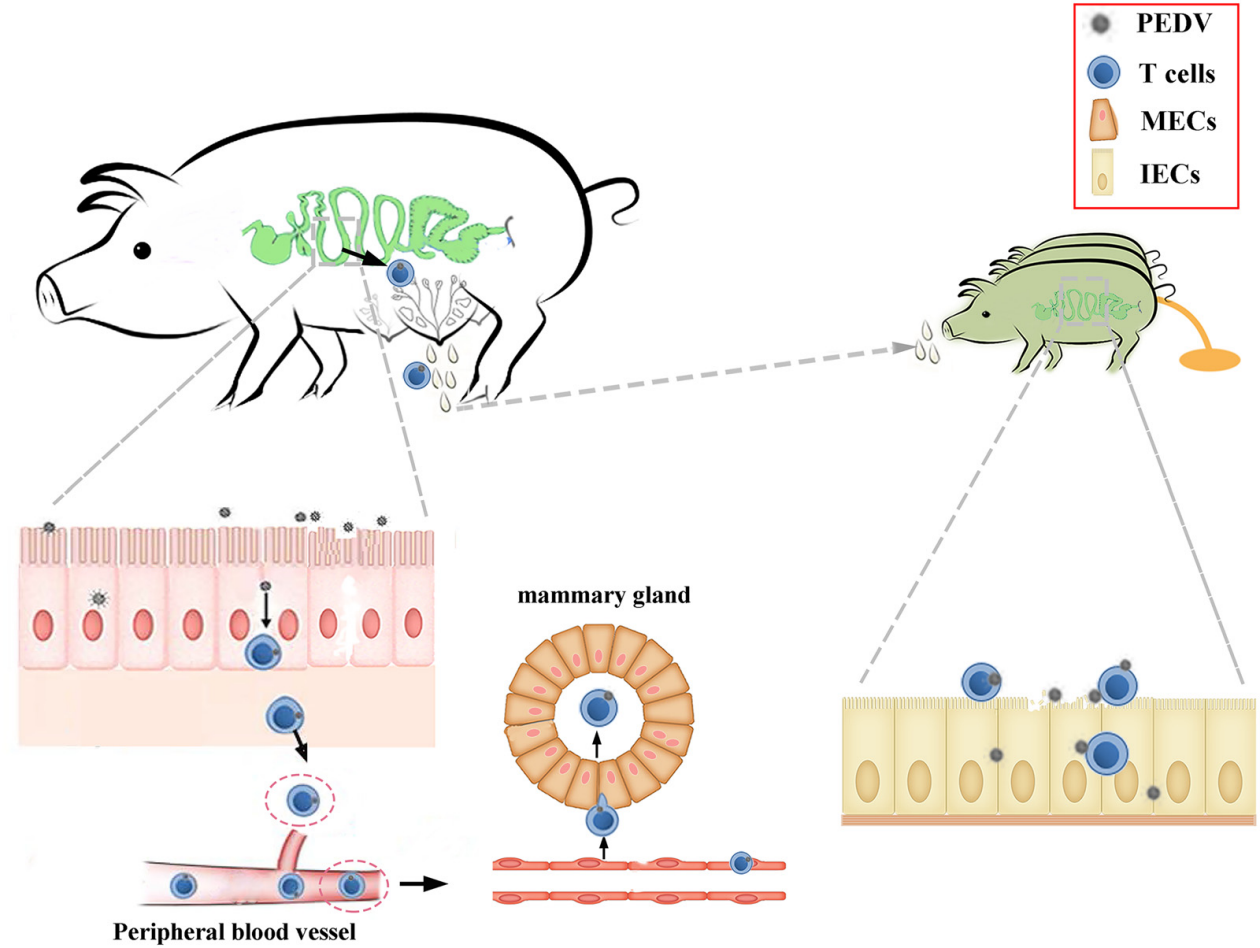
Schematic of the proposed mechanism for the vertical transmission of PEDV from mothers to neonates via breast milk. When the pregnant sows were orally immunized (infected with the wild type), it presents the possibility that sows were infected with PEDV. First, PEDV colonizes intestinal epithelial cells, which allows the virus to be transferred to CD3^+^ T cells located just beneath the epithelium. Second, PEDV-carrying CD3^+^ T cells migrate from the intestine to the mammary gland through the lymph and blood circulation. Finally, the PEDV-carrying CD3^+^ T cells in colostrum can transfer the virus to intestinal epithelial cells via cell-to-cell contact, causing typical PED symptoms in neonatal piglets.

The colostrum represents an important route of infection for many viruses, such as human immunodeficiency virus, Zika virus, and cytomegalovirus (10–12). Recent research shows that severe acute respiratory syndrome coronavirus 2 RNA can be detected in the colostrum (13, 14). Virus transmission results from both cell-free and cell-associated viruses in the colostrum (15). In fact, the presence of cell-associated viruses in the colostrum might be a more significant predictor of neonatal virus infection (16–19). Cell-associated viruses in the colostrum can thus become a major source of infection. Indeed, in our study, PEDV infection in neonatal piglets was mediated by colostrum-derived CD3^+^ T cells. Our study confirmed that PEDV could be transmitted from sows to neonatal piglets via colostrum, and the dissemination of CD3^+^ T cell-associated virus into the colostrum plays an important role in this process (Fig. 9).

PEDV primarily targets intestinal epithelial cells (IECs) after oral immunization of sows. The intestinal mucosal tissue contains a large population of intraepithelial lymphocytes (IELs), which are located beneath or between adjacent IECs (20, 21). IELs can interact and communicate with IECs owing to their close contact (22). IELs can also migrate from the epithelial layer into the lamina propria and vice versa (23, 24). Thus, an interaction between IELs and IECs might be beneficial for the cell-to-cell transfer of viruses. Indeed, PEDV-carrying IECs could transmit the virus to CD3^+^ T cells via cell-to-cell contact. However, the detailed molecular mechanism by which PEDV is transferred from IECs to IELs has not yet been defined.

The differential expression of surface ligands and receptors on lymphocytes and endothelial cells, as well as the expression of chemotactic factors and chemokines, drives lymphocyte migration. Lymphocyte migration from the intestine to the MG is mediated mostly by the interaction between surface integrin α4β7 on lymphocytes and MAdCAM-1 (25–28). HIV-1 virions incorporate integrin α4β7 and can be captured by MAdCAM-1-expressing cells, facilitating HIV-1 infection (29, 30). Moreover, the migratory ability of lymphocytes can be modulated by viruses. This renders infected T cells actively motile, with robust migration, transmitting the virus to target cells and becoming a vital source of infection (31, 32). There was a higher level of integrin α4β7 on CD3^+^ T cells after infection by PEDV, and the expression of MAdCAM-1 in sow colostrum was increased after oral infection with PEDV. Our data suggested that more PEDV-loaded CD3^+^ T cells could migrate to the MG. The migration of lymphocytes depends on cytoskeletal rearrangement. Viruses could use integrin to regulate actin rearrangement, thus promoting infection (33). Similar to those of another study (9), our results showed that PEDV affects actin cytoskeletal rearrangement and cofilin activity via the integrin α4β7 signaling pathway. The transepithelial migration of lymphocytes in the MG of sows is mediated mostly by CCR10 on lymphocytes, interacting with CCL28 (28). There was a higher level of CCR10 on CD3^+^ T cells after infection by PEDV. The levels of CCL28 expression were increased in sow milk after oral infection with PEDV. These data suggested that more CD3^+^ T cells could be easily transported across the mammary epithelium into the lumen during PEDV infection. Additionally, the increase in CCL28 in the MG of swine coincides with the number of accumulating immunoglobulin A (IgA) B cells during gestation (25). Our data suggested that T cell migration, which is consistent with CCL28 controlling IgA B cell accumulation in the lactating MG (34), depends on the interaction between CCR10 on T cells and CCL28 on MECs. Although these *in vitro* studies utilized a variety of epithelial cell lines as *in vitro* models, they do not fully recapitulate the complexity of infection *in vivo*. In this study, an autotransfusion experiment was performed in sows and demonstrated that PEDV-carrying CD3^+^ T cells could migrate to the mammary gland via blood circulation and pass through the mammary epithelium into the lumen. Whether there are other routes for PEDV-carrying CD3^+^ T cells to migrate to the mammary gland is worth exploring further. This was the first evidence to show that virus could influence CD3^+^ T cell trafficking from the blood to the MG *in vivo*.

Through oral inoculation, PEDV-loaded CD3^+^ T cells in the colostrum of immunized infected sows could be inserted between the IECs of neonatal piglets. These results are consistent with those showing that cells in colostrum are absorbed in the digestive tract of neonatal piglets (35). Furthermore, virus transmission occurred between PEDV-loaded CD3^+^ T cells (sow colostrum) and IECs (neonatal piglets). The cell-to-cell transfer of virus is more efficient than cell-free virus transfer and could allow the virus to evade antibody-mediated neutralization (36). On the first day of lactation, more lymphocytes migrate into the MG of sows (37). Thus, cell-associated virus might also be present in higher concentrations in the colostrum than in breast milk since the former is richer in lymphocytes than breast milk (38). Our results showed that the rate of detection of PEDV in sow colostrum was 44.5%, similar to the rate of detection of 40.8% reported in another study (2).

This study not only reveals the specific mechanism underlying the vertical transmission of PEDV via the colostrum but also provides insights into the prevention and control of vertical transmission of other viruses in humans. Moreover, although the colostrum is a major route of PEDV infection in swine farms, avoiding breastfeeding is not an effective way to prevent the vertical transmission of PEDV. Therefore, we suggest that future research should focus on interrupting vertical transmission, avoiding the use of feedback methods, as well as developing PEDV vaccines and antivirals for neonates.

## MATERIALS AND METHODS

### Reagents and cell lines.

Integrin α4β7, integrin α4, and anti-CCR10 were purchased from Novus, MyBioSource, and Bioss, respectively. DyLight 488- and 594-conjugated secondary antibodies were purchased from MultiSciences (Lianke) Biotech Co., Ltd. Anti-pig allophycocyanin (APC)-CD3ε was purchased from BD Biosciences. The anti-PEDV N protein monoclonal antibody (mAb) was purchased from Medgene Labs. The anti-pig epithelial cell marker phycoerythrin (PE)-cytokeratin 18 (CK18) mAb was purchased from Novus Biologicals. TRITC-phalloidin was purchased from Shanghai Yeasen Biotechnology Co., Ltd. Recombinant murine MEC CCL28 was purchased from Peprotech. Phospho-FAK (Tyr397) polyclonal antibody (catalog number PA5-17084) and phospho-cofilin (Ser3) polyclonal antibody (catalog number PA5-17752) were purchased from Invitrogen. ELISA kits (cytokines IL-6, TNF-α, IFN-γ, IL-12, IL-10, IL-4, and TGF-β) were purchased from Jiangsu Meimian Industrial Co., Ltd. CFSE (carboxyfluorescein succinimidyl amino ester) was purchased from Invitrogen. The PKH26 red fluorescent cell linker kit (PKH26GL) was obtained from Sigma-Aldrich. Hoechst 33342 was purchased from Life Technologies. Anti-PE microbeads (catalog number 130-097-054), anti-APC (catalog number 130-097-143), and MiniMACS starting kits were all purchased from Miltenyi Biotec. Vero E6 cells were kindly provided by the Veterinary Medicine Research Center of the Da Bei Nong Group. EpH4-Ev mammary epithelial cells were kindly provided by Jinfeng Miao, Nanjing Agricultural University. The cell line was regularly tested for mycoplasma contamination.

### Animals.

Conventional Duroc × (Landrace × Yorkshire) neonatal piglets and sows were obtained from four swine herds in Jiangxi province, Hebei province, and Jiangsu province. Sows on different farms were fed infected material at different times. Generally, after a PEDV outbreak on a pig farm, all sows, including gilts, are fed infected material at one time, except for sows in the farrowing house (within 21 days before delivery). The neonatal piglets were born via natural farrow and were fed synthetic milk or sow colostrum. Each experimental group of neonatal piglets was housed in a separate room with constant humidity and temperature and a 12-h-light/dark cycle. All procedures and experiments performed on the animals were approved by the Institutional Animal Care and Use Committee of Nanjing Agricultural University and followed National Institutes of Health guidelines for the performance of animal experiments. The animal protocol was approved by Nanjing Agriculture University Committee on Animal Resources Committee (permit number SYXK2011-0036).

### Virus.

The wild-type PEDV strain Zhejiang08 was preserved by our laboratory, which was clustered with the emerging virulent strain.

### Generation of milk cells.

Twenty milliliters of colostrum was collected manually and used to isolate cells. The samples were first filtered through filter membrane (210 mm), and the filtrates were then centrifuged at 340 × *g* for 15 min at 4°C. The supernatant with the fat layer was discarded, the cell pellets were resuspended in 25 mL of phosphate-buffered saline (PBS), and the procedure was repeated. The final cell pellet was then carefully resuspended with a Pasteur pipette in 5 mL PBS. To acquire T cells, the colostrum cells were labeled with APC-CD3 antibody, incubated with anti-APC microbeads, and sorted using MiniMACS starting kits. Next, the sorted CD3^+^ T cells were activated by phytohemagglutinin (PHA) and IL-2 for 3 days for PEDV infection (multiplicity of infection [MOI] = 0.1).

### PEDV oral inoculation.

Neonatal piglets (0 days old) with similar weights were allocated into 2 groups (3 piglets per group) with a completely random design and housed in two separate rooms. Neonatal piglets were selected from immunized infected sows. The neonatal piglets were doubly negative for PEDV and its antibodies. Sows and piglets were kept separately after challenge. The two groups were the controls (group I) and piglets administered colostral CD3^+^ T cells isolated from immunized infected sows (group II). Neonatal piglets absorbed colostrum from their own mother. Neonatal piglets in group II were challenged with 1 mL colostral CD3^+^ T cells by oral inoculation. In group I, the same volume of PBS was inoculated orally as a negative control. The animals were artificially fed colostrum from their own mother every 3 h throughout the experiment to meet or exceed National Research Council (NRC, 2012) requirements for nutrients and energy for piglets of this size. After a challenge, the neonatal piglets were observed daily for symptoms of diarrhea.

### Generation of T cells.

Porcine PBMCs were isolated from the blood of pigs by density centrifugation using a porcine peripheral blood lymphocyte separation kit (Solarbio). The method for isolating lymphocytes from mesenteric lymph nodes (MLNs) is as follows. After the removal of residual mesenteric fat tissue, the lymph node was then cut into 0.5-cm pieces. The pieces were incubated in 20 mL of 10 mM EDTA in Hanks’ balanced salt solution (HBSS) for 20 min at 4°C. The mixtures were then centrifuged, and precipitates were placed into a digestion solution containing 1% fetal bovine serum (FBS), 2 mg/mL each of collagenase D (Roche) and DNase I (Sigma), and 100 U/mL Dispase (Fisher) at 37°C for 20 min with slow rotation. The supernatants were obtained by density gradient centrifugation. To acquire T cells, PBMCs and lymphocytes from MLNs were labeled with APC-CD3 antibody, incubated with anti-APC microbeads, and sorted using MiniMACS starting kits. Next, the sorted CD3^+^ T cells were activated by PHA and IL-2 (Proceth) for 3 days for subsequent experiments.

### PEDV infection and transmission.

Vero cells were infected with PEDV (MOI = 1) for 1 h at 37°C and washed extensively to remove unbound virus. Vero cells infected with PEDV were cocultured with positive CD3^+^ T cells selected from MLNs by two methods (contact and noncontact) that had been maintained at 37°C in RPMI 1640 medium supplemented with PHA and IL-2 for the subsequent study. After 1 h or 6 h, PEDV-carrying CD3^+^ T cells were collected for viral RNA titer determination by RT-PCR and plaque assays. Protein expression of PEDV in CD3^+^ T cells of the coculture system was detected by Western blotting.

### *In vivo* reinfusion experiments.

For the *in vivo* reinfusion assay, CD3^+^ T cells were isolated from PBMCs and infected with PEDV for 3 h. Next, the CD3^+^ T cells infected with PEDV were labeled with CFSE (green) for long-term cell labeling. PEDV-loaded CD3^+^ T cells (5 × 10^7^) labeled with CFSE were then adoptively transferred into a sow (autologously) via ear vein injection. The recipient sow was sacrificed 24 h after the injection. For the flow cytometry analyses, cells were isolated from milk at different times. For histological analyses, the tissues of the MG, including the base area of the mammary gland (AB), the central area of the upper body of the gland (CAUB), and the area surrounding the gland cistern (ASGC), were washed with 0.1 M PBS (pH 7.4) and embedded in OCT compound (Sakura Finetechnical). Frozen tissues of the AB, CAUB, and ASGC from the MG were cut into 8-μm sections and mounted onto poly-l-lysine-coated glass slides. CFSE-labeled cells were observed with a fluorescence microscope (Carl Zeiss).

### Migration on Matrigel.

The lower sides of 8-μm-pore-size polycarbonate filters of a 24-well transwell plate (Corning Incorporated Costar, Cambridge, MA, USA) were coated with 100 μL of 50% Matrigel (BD Biosciences) and allowed to solidify for 20 min at 37°C. A total of 10^6^ blood-derived CD3^+^ cells/mL were labeled with CFSE, infected with PEDV, and seeded onto Matrigel. Medium containing MAdCAM-1 (500 ng/mL) was added to the bottom chamber. Blood-derived CD3^+^ cells were incubated for 24 h at 37°C with 5% CO_2_. Subsequently, the 8-μm-pore-size polycarbonate filters were placed on a slide and observed. Images were taken using a Zeiss LSM710 confocal microscope.

### Transmigration assay.

The lower sides of 5-μm-pore-size polycarbonate filters of a 24-well transwell plate (Corning Incorporated Costar, Cambridge, MA, USA) were seeded with mouse mammary epithelial cells (MECs). The confluence of the MEC monolayers was assessed by measuring their transepithelial electrical resistance (TEER) and by staining tight junction proteins such as ZO-1 with anti-ZO-1 (Invitrogen). Lymphocytes labeled with CFSE were seeded into the upper chamber of MECs. The recombinant chemokine CCL28 (500 ng/mL) and milk from the control or infected group were added to the lower chamber of some filters. After 24 h of migration at 37°C with 5% CO_2_, T cells were collected by centrifugation of the lower-chamber medium and quantified by FACS. The cells covering the filters following migration were observed by fluorescence microscopy. Images were taken using a Zeiss LSM710 confocal microscope.

### Confocal laser scanning immunofluorescence microscopy.

CD3^+^ T cells were infected with PEDV (MOI = 0.1) at different times. The cells were fixed with 4% paraformaldehyde for 15 min and permeabilized with 0.1% Triton X-100 for 5 min. Next, the cells were blocked with 5% bull serum albumin (BSA) for 1 h, and stained cells were incubated with CD3 antibodies overnight at 4°C. After rinsing in PBS, the cells incubated with Alexa Fluor 647-labeled secondary antibodies were kept at room temperature for 1 h. Finally, the cells were stained with 200 nM TRITC-phalloidin–PBS for 40 min and 1 mg/mL 4′,6-diamidino-2-phenylindole (DAPI)–PBS for 10 min to label F-actin and nuclei, respectively. Fluorescence images were then recorded using a confocal laser scanning microscope.

### Immunohistochemistry.

The pigs were anesthetized and sacrificed via intravenous injection of pentobarbital sodium (100 mg/kg of body weight). The duodenum, ileum, and jejunum were taken, and the fractions were subsequently selected for assessing the distribution of PEDV by immunohistochemistry (IHC) using primary antibodies directed against the PEDV N protein. After blocking with 5% bull serum albumin for 1 h, the sections were subsequently incubated with mouse anti-pig PEDV (1:100) overnight at 4°C and then incubated with biotinylated rabbit anti-mouse IgG for 1 h at room temperature. After staining with Diaminobenzidine (DAB), the sections were then sealed with neutral balata. The sections were visualized using a light microscope (CX23; Olympus Corporation, Tokyo, Japan) at a magnification of ×100 or ×400.

### IFA.

Fixed sections were permeabilized in 0.2% Triton X-100 in PBS for 5 min. After washing with PBS 3 times, the filters were blocked with 5% bull serum albumin for 1 h. The filters were incubated with primary antibodies overnight at 4°C, followed by fluorescent secondary antibodies at room temperature for 1 h. The fractions were immunolabeled with goat CD3 mAb and mouse anti-PEDV mAb, followed by Alexa Fluor 594-conjugated rabbit anti-goat IgG and Alexa Fluor 488-conjugated goat anti-mouse IgG.

### Western blot analysis.

Total protein from different tissues or cells, lysed using lysis buffer, was separated by SDS-PAGE and transferred to a polyvinylidene membrane (Millipore). Membranes were blocked with Tris-buffered saline containing 5% BSA and 0.1% Tween 20. Next, the membrane was incubated with the respective primary antibodies, followed by horseradish peroxidase (HRP)-conjugated secondary antibodies in blocking reagent. After extensive washing with Tris-buffered saline–Tween (TBST), immunoreactive bands were analyzed by film exposure after an enhanced chemiluminescence (ECL) reaction.

### Plaque assays.

The viral titers of the supernatant samples were measured by plaque assays. Confluent monolayers of Vero cells grown in 12-well tissue culture plates were infected with 500 μL of serial 10-fold dilutions of the supernatant samples. After incubation for 1 h at 37°C, cells were overlaid with 0.7% agarose in Dulbecco’s modified Eagle’s medium (DMEM) containing 2% FBS and incubated at 37°C. At 3 days postinfection, plaques were visualized by staining with crystal violet.

### Flow cytometric analysis.

The cells were acquired from the different mucosae and stained with the indicated antibodies. After surface staining, the cells were resuspended in a fixation/permeabilization solution (BD Cytofix/Cytoperm kit; BD Pharmingen) for 40 min at 4°C and stained with PEDV N protein antibody to detect intracellular PEDV. After three washes with PBS, the cells were phenotypically analyzed by FACS (BD FACSCalibur).

### Cytokine assays by enzyme-linked immunosorbent assays.

The levels of cytokines in the milk were analyzed by enzyme-linked immunosorbent assays (ELISAs), and PEDV-specific IgA in milk and PEDV-specific IgG in serum were detected by an ELISA. The production of the cytokines IL-6, TNF-α, IFN-γ, IL-12, IL-10, IL-4, and TGF-β was measured using an ELISA kit according to the manufacturer’s instructions.

### RT-qPCR.

Total RNA from different tissues was purified using an RNAiso plus kit (TaKaRa, Dalian, China) according to the manufacturer’s instructions. Fresh RNA (500 ng) was used as a template for the synthesis of the first-strand cDNA with commercial oligo(dT) primers using a PrimeScript II first-strand cDNA synthesis kit (TaKaRa, Dalian, China). PCR was performed with a SYBR green qPCR kit (TaKaRa) using the Applied Biosystems 7500 fast real-time PCR system (Life Technologies). A separate glyceraldehyde-3-phosphate dehydrogenase (GAPDH) amplification was used to normalize gene expression. The data were analyzed using the 2^−ΔΔ^*^CT^* method.

### Statistical analysis.

Results are expressed as the means ± standard deviations (SD) and were analyzed with SPSS 17.0. One-way analysis of variance (ANOVA) was employed to determine significant differences among multiple groups, and a *t* test was employed to determine the differences between two groups (*, *P < *0.05; **, *P < *0.01). Data were combined from at least three independent experiments unless otherwise stated.
